# Analysis of imidazoles and triazoles in biological samples after MicroExtraction by packed sorbent

**DOI:** 10.1080/14756366.2017.1354858

**Published:** 2017-08-04

**Authors:** Cristina Campestre, Marcello Locatelli, Paolo Guglielmi, Elisa De Luca, Giuseppe Bellagamba, Sergio Menta, Gokhan Zengin, Christian Celia, Luisa Di Marzio, Simone Carradori

**Affiliations:** a Department of Pharmacy, University of Chieti – Pescara “G. d’Annunzio”, Chieti, Italy;; b Interuniversity Consortium of Structural and Systems Biology, Rome, Italy;; c Dipartimento di Chimica e Tecnologie del Farmaco, Sapienza University of Rome, Rome, Italy;; d Department of Biology, Selcuk University, Konya, Turkey;; e Inter-Regional Research Center for Food Safety & Health, University of Catanzaro “Magna Græcia”, Catanzaro, Italy;; f Department of Nanomedicine, Houston Methodist Research Institute, Houston, TX, USA

**Keywords:** MEPS-HPLC-DAD, method development, plasma and urine, sample preparation, azole antifungal drugs

## Abstract

This paper reports the MEPS-HPLC-DAD method for the simultaneous determination of 12 azole drugs (bifonazole, butoconazole, clotrimazole, econazole, itraconazole, ketoconazole, miconazole, posaconazole, ravuconazole, terconazole, tioconazole and voriconazole) administered to treat different systemic and topical fungal infections, in biological samples. Azole drugs separation was performed in 36 min. The analytical method was validated in the ranges as follows: 0.02–5 μg mL^−1^ for ravuconazole; 0.2–5 μg mL^−1^ for terconazole; 0.05–5 μg mL^−1^ for the other compounds. Human plasma and urine were used as biological samples during the analysis, while benzyl-4-hydroxybenzoate was used as an internal standard. The precision (RSD%) and trueness (Bias%) values fulfill with International Guidelines requirements. To the best of our knowledge, this is the first HPLC-DAD procedure coupled to MEPS, which provides the simultaneous analysis of 12 azole drugs, available in the market, in human plasma and urine. Moreover, the method was successfully applied for the quantitative determination of two model drugs (itraconazole and miconazole) after oral administration in real samples.

## Graphical abstract

**Figure:**
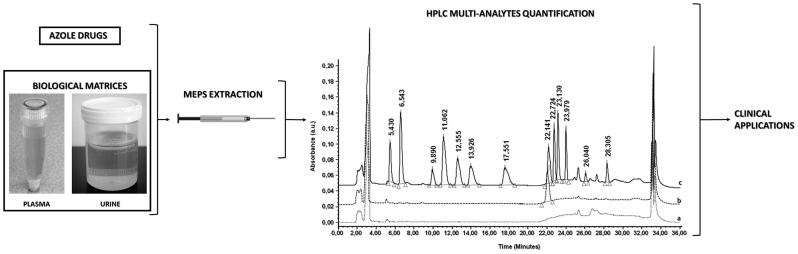

## Introduction

Imidazole drugs are organic water-soluble compounds showing a diazole aromatic heterocycle with non-adjacent nitrogen atoms, which has two equivalent tautomeric forms due to the presence of two nitrogen atoms in the backbone structure. The imidazole ring is widely present in various natural products, e.g. alkaloids, histamine, histidine, purine, which share the same 1,3-C_3_N_2_ basic ring with different substituents on the side chains. The imidazole also forms the key building block of different drugs possessing several biological activities, such as antibacterial, anticancer, antifungal and analgesic^1^.

Triazoles represent another group of azole derivatives with a broader antifungal spectrum and safer profile than imidazoles^2^. The triazole antifungal drugs include fluconazole, isavuconazole, itraconazole, posaconazole, pramiconazole, ravuconazole and voriconazole. Azoles (both imidazoles and triazoles) represent the active principle ingredient (API) of different drugs, which are topically or systemically administered as creams, shampoos, powders, tablets and capsules to treat fungal infections^3^. Bifonazole, butoconazole, clotrimazole, econazole, ketoconazole, miconazole, tioconazole and terconazole are commonly formulated as creams and powders, whereas itraconazole, ketoconazole, posaconazole and voriconazole are formulated as tablets and capsules.

Pursuing our interest in the search and development of antifungal drugs^4–10^, the aim of this project was the quantification of azole derivatives, currently available in the market, in real samples through innovative microextraction techniques.

The analysis of the antifungal drugs in biological samples, e.g. plasma and urine, was usually carried out using high-performance liquid chromatography (HPLC) coupled to fluorimetric detector^11–13^, UV/Vis^14–18^, mass spectrometry (HPLC-MS)^19^, tandem mass spectrometry (HPLC-MS/MS)^20–22^ and other highly hyphenated instrument configurations^23^. HPLC-diode array (DAD) or UV/Vis detectors are also used to quantify azoles in pharmaceutical products^24–26^. A C_18_, C_8_ or C_6_-phenyl stationary phase and acetonitrile, as mobile phase, are often used to set up a rugged and routinary analytical method, which can be scaled up for the industrial quality control procedures.

**Figure 1.**
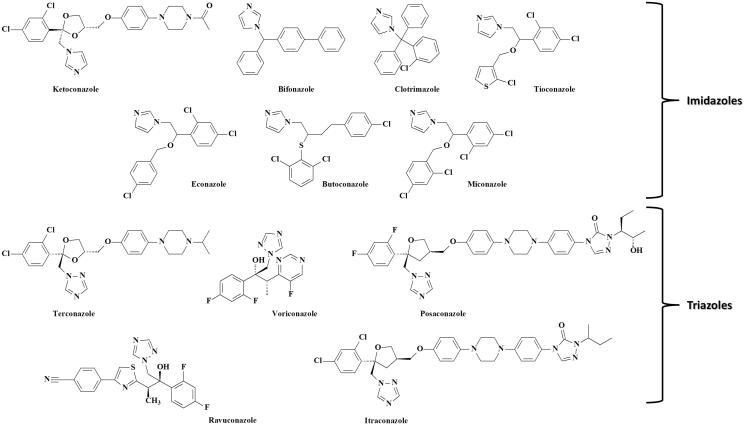
Chemical structures of the selected 12 azoles.

Although several analytical methods were developed to quantitate azole drugs in pharmaceutical compounds, no HPLC-DAD method coupled to microextraction by packed sorbent (MEPS) technique is actually available to analyze simultaneously several azole derivatives in biological samples. Indeed, few works showed the separation of a limited number of triazoles, e.g. posaconazole and voriconazole^15^ ^,^ ^27^, or fluconazole, itraconazole, voriconazole, posaconazole^23^, or fluconazole, itraconazole, voriconazole, posaconazole and ketoconazole^28^, or ketoconazole, tioconazole, econazole, miconazole and itraconazole^29^ from biological samples. Up to date, a proper method for the simultaneous quantification of all the derivatives in Figure 1, suitable for clinical applications, was not reported.

The clean-up and extraction procedure can decrease the recovery of compounds, which show different physical–chemical properties and limit the optimization of the method for multiple drugs determination. The liquid–liquid extraction (LLE) with organic solvents^15^ ^,^ ^27^ ^,^ ^29^ and protein precipitation are widely used to extract drugs from biological samples^30^; unfortunately, they often show suitable performances on a limited number of drugs as demonstrated by the low quantitative recovery of bifonazole, butoconazole, ravuconazole and terconazole in the biological samples. To improve the extraction procedure, the solid phase extraction (SPE) procedure was proposed^28^. In the herein reported study, the SPE extraction was carried out testing different stationary phases, such as Oasis HLB, Strata-X and Sep-Pak. The SPE sorbents were conditioned according to the instructions from the manufacturer and to Gordien et al.’s protocol^28^. The SPE extraction increased drugs recovery (>60%). Unfortunately, for this procedure, a large amount of samples, buffers and organic solvents was required. Furthermore, the SPE procedure could be time-consuming and samples must be dried to reduce volume before HPLC analysis to achieve better LOQ values and to improve the signal-to-noise ratio. To overcome these drawbacks, MEPS microextraction technique was tested to optimize the recovery and the time of analysis, as previously observed in other works^31–33^, and then applied to biological samples. In fact, this procedure increased the recovery of samples and decreased both the time of analysis and the use of organic solvents compared with SPE.

The obtained MEPS-HPLC-DAD protocol can provide several advantages for the concurrent extraction and analysis of azole derivatives in biological samples compared with other complex methods. Furthermore, the availability of a procedure for the determination of several analytes could be useful for clinical applications. The reported method showed comparable (or better) analytical performances to the others reported in the literature (see section Comparisons with existing methods).

## Materials and methods

### Chemicals, solvents and devices

Bifonazole, butoconazole, clotrimazole, econazole, itraconazole, ketoconazole, miconazole, posaconazole, ravuconazole, terconazole, tioconazole, voriconazole and benzyl-4-hydroxybenzoate (Internal Standard, IS) (>98% purity grade), sodium phosphate monobasic and sodium phosphate dibasic (>99% purity grade), phosphoric acid (90% purity grade) and trichloroacetic acid (>99% purity grade) were purchased from Sigma-Aldrich (Milan, Italy).

The commercial drugs were obtained from a local Pharmacy after medical prescription. Both HPLC-grade Acetonitrile (AcN) and methanol (MeOH) were supplied by Carlo Erba (Milan, Italy) and were used without further purification. The water for HPLC analysis was generated by using a Millipore Milli-Q Plus water treatment system (Millipore Bedford Corp., Bedford, MA). Oasis HLB (1 mL, 30 mg) and Sep-Pak (1 mL, 50 mg) were obtained from Waters (Milford, MA), while Strata-X (1 mL, 30 mg) was purchased from Phenomenex (Torrance, CA). MEPS device (syringe) and replacement needle with C_18_ stationary phase were purchased from SGE Analytical Science (Melbourne, Australia).

### Stock solution, calibration curve and quality control analysis

The 12 stock solutions of chemical standards were made at the concentration of 1 mg mL^−1^ in the mobile phase. The combined working solutions (concentration range: 0.5–50 μg mL^−1^) were carried out by dilution of a mixed solution. The matrix-matched calibration standards and quality control samples (QCs) were carried out in biological matrices (plasma and urine) as reported in Section “Plasma and urine samples preparation”. The resulting samples were injected in HPLC-DAD instrument after MEPS extraction.

### Plasma and urine collection and storage

Plasma and urine samples were collected from healthy volunteers, which were informed on the experimental procedures and the nature of the study, and gave a written consent. No medications were administered to healthy volunteers before the experiments. The study was conducted in line with Good Clinical Practice guidelines and with the ethical principles laid down in the latest version of the Declaration of Helsinki. The plasma samples were collected into heparinized tubes, while urine samples were collected into sterilized containers. All samples were stored at −20 °C before the analysis.

### Plasma and urine samples preparation

About 170 μL of human blank plasma or urine were mixed with 20 μL of analyte working solutions and 10 μL of IS (1 μg mL^−1^) and vortexed for 3 min (15% v:v of matrix modification for calibration and QC samples and 5% v:v of matrix modification for real samples).

Biological samples were diluted using trichloroacetic acid (TCA) (20 mg mL^−1^) in 1:0.5 (v:v) ratio^31–33^, centrifuged (12,000 × *g* for 10 min) and extracted by MEPS apparatus. The TCA treatment was carried out to denature the biological proteins, to hydrolyze the drug-protein binding and to reduce the sample density^34^ (widely used in biochemistry also for the precipitation of macromolecules, such as proteins, DNA and RNA). This acid was chosen to provide greater stability of the selected analytes in acidic conditions with respect to the reported harsh conditions (1.0 M perchloric acid)^17^. The supernatant was loaded in the MEPS, cleaned using water (200 μL), eluted and directly injected into the HPLC-DAD using methanol (20 μL). The analysis showed the presence of interference peaks, but a suitable recovery of drugs in terms of signal-to-noise ratio and peak areas compared with SPE treated samples.

**Figure 2.**
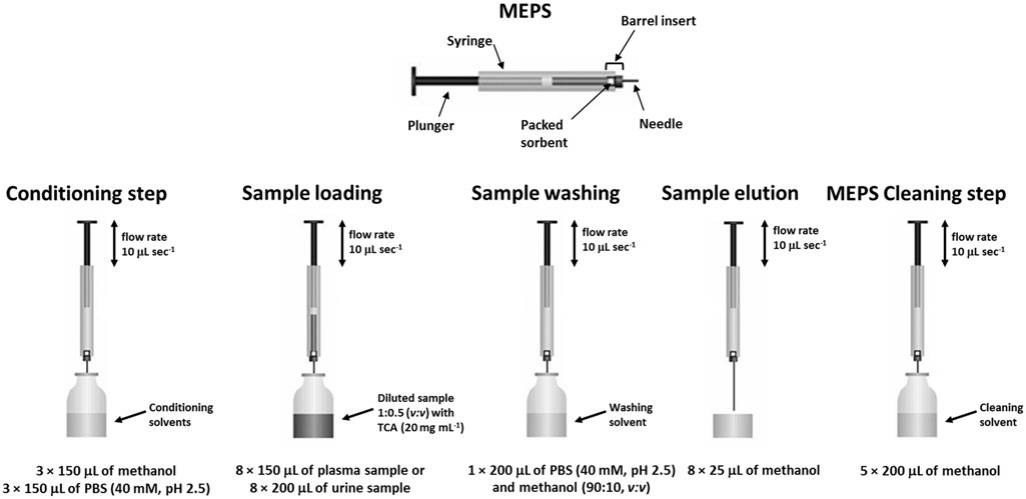
Schematic illustration of the optimized MEPS extraction procedure.

The *off-line* extraction procedure was optimized as follows: (i) conditioning of sorbent with 3 × 150 μL of methanol and 3 × 150 μL of phosphate buffer (40 mM, pH 2.5); (ii) loading of plasma samples diluted 1:0.5 (v:v) with TCA (20 mg mL^−1^) (8 × 150 μL) or urine samples diluted 1:0.5 (v:v) with TCA (20 mg mL^−1^) (8 × 200 μL); (iii) washing with 1 × 200 μL of phosphate buffer (40 mM, pH 2.5) and methanol (90:10, v:v); (iv) elution of samples with 8 × 25 μL of methanol in a single vial by using an average flow rate of 10 μL s^−1^, and then directly injected into the HPLC-DAD system. This optimized procedure is reported graphically in Figure 2.

### Apparatus and chromatographic conditions

Analyses were performed using an HPLC Waters liquid chromatography (model 600 solvent pump, 2996 DAD, Waters Corporation, Milford, MA). Mobile phase was directly *on-line* degassed by using a Biotech 4CH DEGASI Compact (Biotech Inc., Onsala, Sweden). Empower v0.2 Software (Waters Spa, Milford, MA) was used to collect and analyze the data.

Two different columns were used to optimize the chromatographic conditions: Luna C_18_ (250 × 4.6 mm, 5 μm particle size, Phenomenex, Torrance, CA) and Discovery C_8_ column (250 × 4.6 mm, 5 μm particle size, Supelco, Milan, Italy). The Luna C_18_ packing column connected to a Security Guard column (4.0 × 3.0 mm, 5 μm particle size; Phenomenex, Torrance, CA) was finally used to separate 12 azole drugs and IS. The columns were thermostated at 25 °C (± 1 °C) using a Jetstream2 Plus column oven during the analysis.

The maximum wavelength of 210 nm^24^ was used to obtain the best signal-to-noise ratio during HPLC-DAD analysis and the maximum values of LODs, LOQs, although these compounds can be detected at 250 nm^25^, 243 nm and 220 nm^35^. These secondary maximum wavelengths were used to further identify the azole drugs during the analysis (Supplementary material, Section S1 for analytes and IS UV/Vis spectra and Section S2 for system suitability test).

The HPLC system was optimized to set-up the better signal-to-noise ratio of drugs in a single chromatographic analysis, the best peak shape, an appropriate run-time and the better peak resolution. First, the analyses were performed in isocratic conditions using Luna C_18_ or Discovery C_8_ column as previously reported^16^ ^,^ ^19^ ^,^ ^27^, in different mobile phases, made up from organic solvents and buffers with increasing ionic strength (15 mM, 30 mM, 40 mM and 50 mM), in different temperature (25 °C, 35 °C and 40 °C) and pH (2.5, 3.0 and 5.5) conditions. The flow rate was always kept at 1.0 mL min^−1^ during the analysis.

Results showed that the retention times decreased by increasing the buffer ionic strength from 15 mM to 30 mM phosphate buffer (pH 2.5). The retention times of drugs were also reduced by increasing column temperature from 25 °C to 40 °C; unfortunately, a broader shape of peaks occurred, probably related to an increased mass transfer kinetics and a corresponding reduction in terms of resolution and peak symmetry. Similar results were obtained by increasing pH value from 2.5 to 5.5. Symmetric peaks were obtained both at 40 mM and 50 mM of phosphate buffer (pH 2.5). A suitable resolution was obtained using Luna C_18_, phosphate buffer (40 mM, pH 2.5) as solvent A, acetonitrile as solvent B, isocratic conditions (58:42, v:v) at 25 °C.

**Figure 3.**
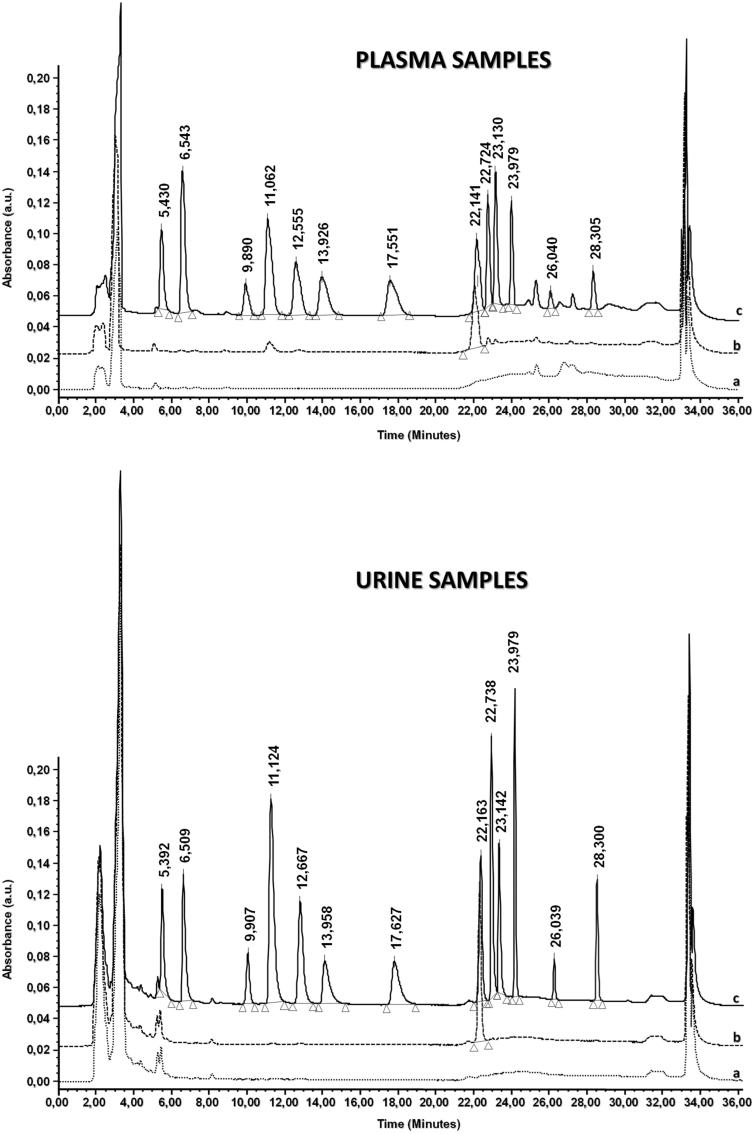
Chromatograms obtained after the extraction and analysis of 12 azoles and benzyl-4-hydroxybenzoate (IS) at the wavelength of 210 nm, respectively (up, plasma sample and, down, urine sample: (a) blank sample, (b) blank sample spiked with 5 μg mL^−1^ of IS and (c) blank sample spiked with 5 μg mL^−1^ of IS and 4 μg mL^−1^ of different drugs). 20 μL of samples were injected during the analysis.

This condition provided the simultaneous analysis of the 12 azole drugs, but the major drawback was the high-retention times of ravuconazole (61.8 min) and itraconazole (67.2 min). The long-retention times of these two drugs in addition to the run time analysis (>68 min) and the low signal-to-noise ratio hampered the isocratic condition preliminary performed. For these reasons, different gradient elution modes, at the same conditions of ionic strength, pH and temperature, were tested to decrease the run time and improve the signal-to-noise ratio. The optimized gradient elution was 0–16 min, 42% B; 16–18 min, from 42% to 70% B, linear; 18–21 min, from 70% to 80% B, linear; 21–28 min, 80% B; 28–36 min, 42% B. This gradient allowed separating simultaneously these drugs and IS without any overlapping and interferences during the analysis (Figure 3). By using the optimized separation conditions, a baseline resolution for different drugs and IS was carried out by 36 min and the resulting retention times are reported in Tables 1 and 2 (see also Supplementary material, Section S2 for System Suitability Test (SST) separation).

**Table 1.** Mean linear calibration curve parameters performed by weighted-linear least-squares regression analysis of six independent eight non-zero concentration points in plasma samples.

	Linearity range	Slope^a^		Intercept^a^		Determination	Wavelength	Retention time (min)	
		Analyte	(μg mL^−1^)	Mean	Std. dev.			Mean	Std. dev.	coefficient (*r*^2^)	(nm)	Mean	Std. dev.
Ketoconazole	0.05–5 (0.017 μg mL^−1^)^b^	0.07762	±0.00485	−0.00211	±0.00019	0.9846		5.49	±0.12
Terconazole	0.2–5 (0.070 μg mL^−1^)^b^	0.1906	±0.016364	0.01732	±0.00207	0.9954		6.63	±0.14
Voriconazole	0.05–5 (0.017 μg mL^−1^)^b^	0.084313	±0.00568	0.002999	±0.002959	0.9917		9.94	±0.13
Bifonazole	0.05–5 (0.017 μg mL^−1^)^b^	0.376833	±0.036575	0.01551	±0.002246	0.9910		11.23	±0.30
Clotrimazole	0.05–5 (0.017 μg mL^−1^)^b^	0.1885	±0.015932	0.01477	±0.001127	0.9961		12.75	±0.45
Tioconazole	0.05–5 (0.017 μg mL^−1^)^b^	0.113067	±0.014298	0.005134	±0.000549	0.9908	210	14.19	±0.34
Econazole	0.05–5 (0.017 μg mL^−1^)^b^	0.129033	±0.014347	0.003792	±0.002456	0.9927		17.84	±0.45
Butoconazole	0.05–5 (0.017 μg mL^−1^)^b^	0.2452	±0.022889	0.002835	±0.004686	0.9925		22.64	±1.84
Miconazole	0.05–5 (0.017 μg mL^−1^)^b^	0.129033	±0.020994	−0.00083	±0.003097	0.9922		23.22	±0.12
Posaconazole	0.05–5 (0.017 μg mL^−1^)^b^	0.1483	±0.021392	−0.00155	±0.001385	0.9962		24.01	±0.09
Ravuconazole	0.02–5 (0.007 μg mL^−1^)^b^	0.032007	±0.002906	0.002364	±0.000659	0.9902		26.06	±0.11
Itraconazole	0.05–5 (0.017 μg mL^−1^)^b^	0.059923	±0.011036	0.015467	±0.002012	0.9924		28.31	±0.10

a Values at 95% confidence intervals on the mean of six independent calibration curves.

b The round brackets show the LOD values obtained from signal-to-noise ratio^3^; the slope and intercept of calibration curve are expressed in μg mL^−1^.

**Table 2.** Mean linear calibration curve parameters performed by weighted-linear least-squares regression analysis of six independent eight non-zero concentration points in urine samples.

	Linearity range	Slope^a^		Intercept^a^		Determination	Wavelength	Retention time (min)	
		Analyte	(μg mL^−1^)	Mean	Std. dev.			Mean	Std. dev.	coefficient (*r*^2^)	(nm)	Mean	Std. dev.
Ketoconazole	0.05–5 (0.017 μg mL^−1^)^b^	0.093827	±0.005462	−0.00211	±0.00019	0.9877		5.49	±0.12
Terconazole	0.2–5 (0.070 μg mL^−1^)^b^	0.182667	±0.019981	0.01732	±0.00207	0.9938		6.63	±0.14
Voriconazole	0.05–5 (0.017 μg mL^−1^)^b^	0.070917	±0.001684	0.002999	±0.002959	0.9934		9.94	±0.13
Bifonazole	0.05–5 (0.017 μg mL^−1^)^b^	0.384167	±0.082413	0.01551	±0.002246	0.9958		11.23	±0.30
Clotrimazole	0.05–5 (0.017 μg mL^−1^)^b^	0.199733	±0.044602	0.01477	±0.001127	0.9970		12.75	±0.45
Tioconazole	0.05–5 (0.017 μg mL^−1^)^b^	0.1181	±0.018455	0.005134	±0.000549	0.9901	210	14.19	±0.34
Econazole	0.05–5 (0.017 μg mL^−1^)^b^	0.135533	±0.0252	0.003792	±0.002456	0.9870		17.84	±0.45
Butoconazole	0.05–5 (0.017 μg mL^−1^)^b^	0.232067	±0.01817	0.002835	±0.004686	0.9944		22.64	±1.84
Miconazole	0.05–5 (0.017 μg mL^−1^)^b^	0.134333	±0.019278	−0.00083	±0.003097	0.9980		23.22	±0.12
Posaconazole	0.05–5 (0.017 μg mL^−1^)^b^	0.2156	±0.020832	−0.00155	±0.001385	0.9951		24.01	±0.09
Ravuconazole	0.02–5 (0.007 μg mL^−1^)^b^	0.03004	±0.003518	0.002364	±0.000659	0.9969		26.06	±0.11
Itraconazole	0.05–5 (0.017 μg mL^−1^)^b^	0.098023	±0.011686	0.015467	±0.002012	0.9944		28.31	±0.10

a Values at 95% confidence intervals on the mean of six independent calibration curves.

b The round brackets show the LOD values obtained from signal-to-noise ratio^3^; the slope and intercept of calibration curve are expressed in μg mL^−1^.

Summarizing, the optimized HPLC elution comprises mobile phase composed by phosphate buffer (40 mM, pH 2.5), as solvent A, and AcN, as solvent B, at a flow rate of 1.0 mL min^−1^, and the following gradient elution: 0–16 min, 42% B; 16–18 min, from 42% to 70% B, linear; 18–21 min, from 70% to 80% B, linear; 21–28 min, 80% B; 28–36 min, 42% B.

### Method validation

The validation of analytical method was carried out according to the International Guidelines^36–38^ in order to check LODs, LOQs, linearity, intra- and inter-day trueness and precision, selectivity, recovery, stability and parallelism test of different drugs in plasma and urine samples.

The LOQ of the method was defined as the concentration of the lowest standard on the calibration curve for which (a) the analyte peak was identifiable and discrete, (b) the analyte response was at least 10 times the response of the blank sample and (c) the analyte response was reproducible with a precision less than 20% and trueness better of 80–120%. The LOD was estimated at a signal-to-noise ratio of 3:1 by injecting a series of samples with known concentrations. Precision and trueness studies were carried out at the LOQ and at three QC concentration levels by injecting six different preparations of the analytes and calculating the RSD% and Bias% of the back-calculated concentrations. Calibration curves were calculated by analyzing six-times these eight non-zero concentration standards (0.02, 0.05, 0.10, 0.20, 0.50, 0.80, 2.00 and 5.00 μg mL^−1^, for ravuconazole; 0.20, 0.30, 0.50, 0.80, 1.50, 2.00, 3.00 and 5.00 μg mL^−1^ for terconazole; 0.05, 0.10, 0.20, 0.30, 0.50, 0.80, 2.00 and 5.00 μg mL^−1^, for all other azole drugs) prepared in freshly spiked plasma (and urine).

Concentrations of the QCs and unknown samples were calculated by interpolating their analyte peak area/Internal Standard area ratio on the calibration curve. Selectivity was tested by analyzing, under optimized chromatographic conditions, six blank plasma and six urine samples from different sources, and by comparing them with spiked ones at a concentration close to the LOQ.

## Results and discussion

### Optimization of MEPS extraction procedure

Up to date MEPS applications cover a large variety of organic compounds due to a great availability of sorbents (C_2_, C_8_, C_18_, C_8_-SCX, SCX, SAX, silica and molecularly imprinted polymers). For the herein considered analytes and for their chemical structures and a wide range of log *K*_ow_ values (ranging from 1.0 for voriconazole to 6.70 of butoconazole), the C_18_ type could represent a valid starting point for extraction process optimization. The MEPS optimization was performed using the blank samples (plasma and urine) spiked with 0.15 μg mL^−1^ of different azole drugs. Using these samples, the different parameters that could affect the extraction process were evaluated.

#### Effect of sample volume

**Figure 4.**
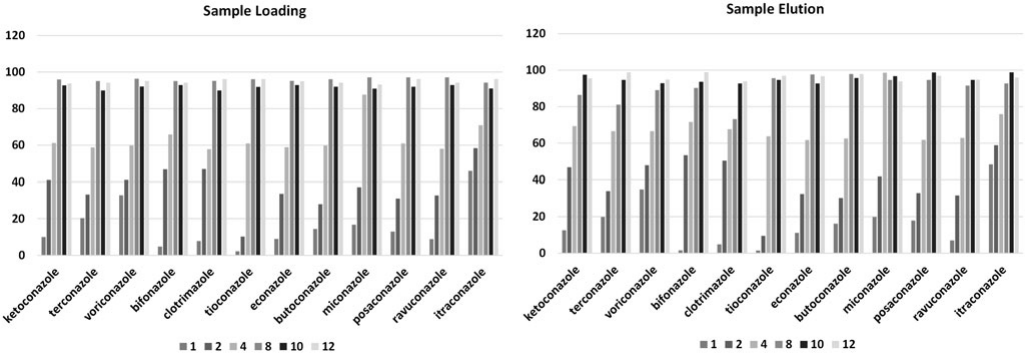
Effects of cycles number on process efficiency for the different analytes during sample loading and sample elution. Values are reported as percentage of observed analyte area respect to a water-based sample spiked at the same concentration level and submitted to the same extraction procedure.

During extraction procedure optimization, especially by using MEPS device, the sample volume covers an important role. In fact, when complex matrices were analyzed, matrix components could saturate the stationary phase, reducing the availability of functional groups that can retain the analyte. Additionally, the use of large sample volume could represent a limiting point for the method applicability, particularly when biological matrices are considered. For these reasons and based on previously validated MEPS procedures^31–33^, the whole extraction process was tested and evaluated on a total sample volume of 150 μL for plasma and 200 μL for urine. Furthermore, in the MEPS procedure, the cycles number could also represent a critical point, due to the possible pre-concentration enhancement particularly during sample loading and sample elution. The effects of cycles number on process efficiency for the different analytes are reported in Figure 4.

#### Effect of type of washing solvent and organic solvent percentage

To optimize the MEPS extraction, two different washing solvent systems were tested: (i) methanol and phosphate buffer (pH 2.5, 40 mM) at the ratios of 5:95 and 10:90 (v:v), (ii) methanol and water with HCl (0.1 N) at the ratios of 5:95 and 10:90 (v:v). The obtained HPLC chromatograms showed that phosphate buffer (pH 2.5, 40 mM) and methanol (90:10, v:v) (1 × 200 μL) were the best performant solvent mixture, because it improved the analytes extraction. The use of MEPS also decreased the time of sample extraction up to 7 min, plus further 10 min to centrifuge samples.

#### Effect of number of draw–eject cycles

The instrument response, in terms of peak area, was optimized, by performing multiple draw–eject extraction cycles and improving the final clean-up of the device, in order also to increase the device lifetime and the number of analyses by a single MEPS needle. The increase of draw–eject extraction cycles improved the peak area for all analytes and reached a plateau after eight cycles (Figure 4). Additionally, due to higher volumes available, for urine samples, the procedure was performed with 200 μL instead of 150 μL. This last value represented the maximum volume that could be obtained after protein precipitation with TCA.

#### Effect of type of eluting solvent and MEPS memory effects

The back extracting solvent volume is another critical parameter that needs to be optimized in order to enhance the signal-to-noise ratio. We tested different methanol volumes, 50, 75, 100, 150, 200 and 300 μL, and observed a signal improvement until 150 μL before reaching a plateau. The methanol was used as an eluent to obtain the maximum recovery of drugs and increased the area of the peak for all compounds and IS.

The carry-over effect was evaluated using blank plasma (or urine) injected after the analysis of fortified biological samples at the upper limit of quantification (ULOQ, 5 μg mL^−1^) and no memory effect of analytical procedure was obtained during the analysis.

#### Selectivity

The selectivity was performed using six blank biological samples collected from different healthy volunteers, according to ICH guidelines^37^. The blank samples showed neither area values over 20% of LOQ areas at the analyte retention times nor over 5% of IS area at the drug retention time. This parameter was evaluated using blank biological samples (plasma and urine) extracted by MEPS device and analyzed with HPLC-DAD without any fortification (a), after fortification with IS (b) or drugs and IS (c) (Figure 3). Results demonstrated that the retention times of drugs were similar to those obtained for real samples and no interfering peaks were detected during the analysis at 210 nm (Figure 3). The peak before the retention time of ketoconazole was observed during the analysis; however, the peak intensity and its retention did not change in all chromatograms and did not affect the ketoconazole quantification both in plasma and urine samples.

#### Effect of type of cleaning solvent on device lifetime

The cleaning solvent adopted in the optimized MEPS procedure is pure methanol due to its high-elution capacity when the C_18_ stationary phase was used. The cleaning solvent choice was guided also by the possible increase in device lifetime and reduction of carry-over phenomena. For these reasons, the MEPS device was washed using pure methanol (5 × 200 μL) before another extraction and no carry-over phenomena were observed. Furthermore, the device could be re-used up to 70–90-folds, without any loss of herein validated analytical performances, by using methanol.

### Method validation

The matrix-matched calibration curves were calculated by analyzing six-times the non-zero concentration standards made in freshly spiked plasma or urine samples. The results were obtained by plotting the analyte/IS area ratio for each level of quantification versus the nominal concentration of each standard solution. The linearity of the method was evaluated by calculating the intercept, slope, determination coefficient and variation in the range 0.02–5 μg mL^−1^ for ravuconazole, 0.2–5 μg mL^−1^ for terconazole and 0.05–5 μg mL^−1^ for other azole drugs (Tables 1 and 2). The different curves were linear over the range reported with coefficients (*r* ^2^) ≥ 0.9846 and weighing factor of (1/*x* ^2^), which are according to International Guidelines^36^. The calibration curve parameters are reported in Table 1 for plasma sample and in Table 2 for urine sample. The LOQ values were 0.02 μg mL^−1^ for ravuconazole, 0.2 μg mL^−1^ for terconazole and 0.05 μg mL^−1^ for other azole drugs. The LOD values were 0.007 μg mL^−1^ for ravuconazole, 0.07 μg mL^−1^ for terconazole and 0.017 μg mL^−1^ for other azole drugs (Tables 1 and 2). The resulting values were calculated at a signal-to-noise ratio (3:1).

The within-assay precision (repeatability) of the same day was carried out by performing six consecutive analyses of QC samples spiked at three different drug concentrations, e.g. 0.4 (low level), 1.0 (medium level) and 4.0 (high level) μg mL^−1^, which are within the calibration curve. The QC samples were also analyzed in different days to evaluate the between-assay precision (intermediate precision). The trueness was tested using the same QC concentrations and comparing the back-calculated concentrations with their nominal values. The intra- and inter-day precision (RSD%) values were ≤13.4% and ≤13.1%, respectively. The intra- and inter-day trueness (Bias%) values were between −13.4% and 12.4% (Supplementary material, Section S3, Table S3.1 for plasma and Table S3.2 for urine). These values strictly respect the limits set requested by the International Guidelines^37^, even if are good and comparable with general analytical procedures, they could be improved by taking to *on-line* MEPS systems, in order to further standardize the extraction process and certainly improve the reproducibility between samples.

The QCs over 50 μg mL^−1^ were also analyzed by MEPS-HPLC-DAD after a dilution (50 folds, v:v) with the corresponding blank matrix. The precision and trueness QC values were comparable with those obtained for low, medium and high drug concentrations. Moreover, the intra-matrix variability was assessed by analyzing different batches of plasma or urine and no interferences were observed. No noteworthy changes of drugs concentrations were observed for stock solutions, working solutions and spiked samples. The spiked samples stored at −20 °C, at +4 °C, and freezing-thawed samples (*n* = 3 cycles) were stable for at least 1 month (Supplementary material, Section S4 for long-term stability, Table S4.1 for plasma and Table S4.2 for urine), as previously reported^15^ ^,^ ^25^ ^,^ ^39^.

The parallelism test was performed using high drug concentrations diluted 50-folds (v:v) with the corresponding blank matrix. Data showed that an upper limit of quantification could be accepted for different samples until 50 μg mL^−1^ and above the maximum value of linearity range reported without decreasing the analytical parameters.

### Comparisons with existing methods

The reported method showed several advantages to analyze simultaneously 12 azole drugs in terms of time-consuming, simplicity and routinary instrumentation, analytical performances, applications in clinic and pharmaceutical fields. Additionally, it shows comparable performances with respect to FPSE-HPLC-PDA procedure reported in the literature^40^. This method also improved the detection and quantification limits for azole drugs in biological samples. Published data showed that few azoles (only up to five drugs) can be simultaneously quantified in biological samples using a single analytical procedure (Table 3). Indeed, different protocols used complex and highly hyphenated configurations^23^ ^,^ ^41^, which require time and solvent consuming for the samples extraction^12^ ^,^ ^27^ ^,^ ^29^ and to obtain low level of LOQs. Other procedures showed that few azoles drugs can be simultaneously analyzed using methods previously reported; but only one protocol^29^ showed the simultaneous analysis of six azoles in cosmetic formulations with a total running time of 80 min and LOQ values similar to those obtained in this study with only 36 min of analysis (Table 3).

**Table 3.** Comparison of published analytical methods for the analysis of azoles extracted from different biological samples.

Sample	Analytes	Extraction	Instrument setting up	Stationary phase	Total extraction procedure time (minutes)	Chromatographic analysis (minutes)	LOQ (μg mL^−1^)	Linear dynamic range (μg mL^−1^)	Ref.
Serum	Posaconazole	Column switching	HPLC-FLD	C_18_	3	17	0.1	0.1–5	^13^
Plasma/saliva	Voriconazole	LLE	HPLC-FLD	C_18_	6	12	0.1	0.1–10	^12^
Plasma	Itraconazole/metabolite	LLE	HPLC-FLD	C_18_	25	30	0.005	0.005–0.5	^11^
Plasma	Itraconazole	Protein precipitation	HPLC-UV/Vis	C_18_	10	–	0.08	–	^14^
Plasma	Voriconazole	LLE	HPLC-UV/Vis	C_18_	15, plus dryness	20	0.1	0.1–20	^15^
	Posaconazole						0.05	0.05**–**10	
Plasma	Posaconazole	–	HPLC-PDA	C_8_	–	65	–	–	^16^
Plasma	Itraconazole	Protein precipitation	HPLC-MS/MS	C_18_	–	–	0.001	0.001–0.5	^20^
Plasma	Voriconazole	LLE	HPLC-MS/MS	C_18_	12	–	0.027	–	^21^
Plasma	Posaconazole	LLE	HPLC-MS/MS	C_18_	–	–	0.001	–	^22^
Interlaboratory Test	Itraconazole	–	–	–	–	–	–	–	^23^
	Voriconazole								
	Posaconazole								
	Fluconazole								
Formulation	Itraconazole	–	HPLC-UV/Vis	Sb-Aq	10	–	–	–	^24^
Formulation	Voriconazole	–	HPLC-UV/Vis	C_18_	–	20	–	–	^25^
	Related impurities								
Ophtalmic	Voriconazole	–	HPLC-UV/Vis	C_18_	–	–	–	–	^26^
Plasma	Voriconazole	LLE	HPLC-UV/Vis	C_8_	25, plus dryness	20	0.2	0.2–10	^27^
	Posaconazole						0.05	0.05**–**10	
Plasma	Fluconazole	SPE	HPLC-UV/Vis	C_6_-phenyl	–	19	0.05	0.05–50	^30^
	Posaconazole							0.05**–**40	
	Voriconazole							0.05**–**40	
	Itraconazole/metabolite							0.05**–**40	
Cosmetic	Ketoconazole	Ultrasound and Liquid Extraction	HPLC-UV/Vis	RP amide C_16_	10, plus centrifugation	80	0.05	0.05–100	^28^
	Tioconazole								
	Econazole								
	Miconazole								
	Itraconazole								
	Clotrimazole								
Plasma	Itraconazole	Protein precipitation	HPLC-MS	C_18_	10	13	0.03	0.03–8	^19^
	Voriconazole						0.03	0.03–8	
	Posaconazole						0.04	0.04–10	
Plasma	Itraconazole	Protein precipitation	HPLC-UV/Vis	C_6_-phenyl	5	17	0.05	0.05–10	^17^
	Voriconazole								
	Posaconazole								
Rat plasma	Itraconazole	LLE	HPLC-DAD	C_18_	12, plus dryness	–	0.05	0.05–5	^18^
	Posaconazole								
Plasma	Posaconazole	Protein precipitation	HPLC-MS/MS	C_18_ and PFP	10	4	0.1	0.1–20	^42^
	Voriconazole								
	Itraconazole/metabolite								
Plasma	Voriconazole	LLE	UPLC-UV/Vis	BEH Phenyl	10, plus dryness	6	0.05	0.05–10	^43^
	Posaconazole								
	Isavuconazole								
	Itraconazole/metabolite								
Plasma	Itraconazole	SLE	UHPLC-MS/MS	Kinetex F5 (PFP)	34	7.6	0.005	0.005–2.5	^39^
	Hydroxy itraconazole								
	Keto itraconazole								
	*N*-Desalkyl itraconazole								
Serum	Voriconazole	Protein precipitation	UFLC-MS/MS	Kinetex C_18_	6	4	0.1	0.1–10	^44^
Plasma/urine	Ketoconazole	MEPS	HPLC-DAD	C_18_	7	36	0.05	0.05–5	Current paper
	Terconazole						0.2	0.2**–**5	
	Voriconazole						0.05	0.05**–**5	
	Bifonazole						0.05	0.05**–**5	
	Clotrimazole						0.05	0.05**–**5	
	Tioconazole						0.05	0.05**–**5	
	Econazole						0.05	0.05**–**5	
	Butoconazole						0.05	0.05**–**5	
	Miconazole						0.05	0.05**–**5	
	Posaconazole						0.05	0.05**–**5	
	Ravuconazole						0.02	0.02**–**5	
	Itraconazole						0.05	0.05–5	

PFP: pentafluorophenyl; RP: reversed phase; LLE: liquid**–**liquid extraction; SLE: solid-supported liquid extraction; plus dryness: required time for complete solvent evaporation is not reported; plus centrifugation: required time for sample centrifugation is not reported.

The LOQ values, which were carried out using MEPS-HPLC-DAD procedure, were similar to those obtained using more sensitive and selective detectors, e.g. FLD^11^ or very expensive MS and MS/MS^19^ ^,^ ^21^ ^,^ ^42^. In many cases, the LOQs of MEPS-HPLC-DAD procedure showed better values (two-folds) than published data, which were obtained using few drugs^12^ ^,^ ^13^ ^,^ ^15^ ^,^ ^27^. The use of benzyl-4-hydroxybenzoate as IS allowed monitoring the efficacy of the extraction and the analytical procedure.

As reported in Table 3, to analyze, a pharmaceutical formulation is necessary to validate a large linear dynamic range (e.g. from 0.05 to 100 μg mL^−1^)^29^, while this necessity is not mandatory when biological matrices are considered. In fact, the availability of a large dynamic range could help in reducing the number of over-range samples that need to be re-analyzed.

For azoles herein considered, the biological levels generally observed are close from low μg mL^−1^ to medium ng mL^−1^ range^17–19^ for plasma, while for urine, it is necessary to validate low ng mL^−1^ range due to the low percentage of unchanged analyte. For this reason, the use of large dynamic range is not necessary as well as the availability of a high sensitivity in biological matrix analyses. Additionally, the use of large range could affect the method accuracy (precision and trueness) at low concentration levels.

### Application to real plasma and urine samples

The performances of analytical method were tested in plasma and urine samples collected from healthy volunteers after single oral administration of commercial capsules of itraconazole (100 mg) and tablets of micozanole (500 mg). Biological samples were extracted by MEPS device and quantified using HPLC-DAD according to the reported validated method. Table 4 reports the quantitative data obtained after real samples analyses (Supplementary material, Section S5 for real samples chromatograms).

**Table 4.** Quantitative analysis of plasma or urine samples collected from healthy human volunteers after single oral dose of commercial capsules of itraconazole (100 mg) and commercial tablets of miconazole (500 mg). Plasma was collected 4 h after the oral administration of drugs, while urine samples were collected at different times.

						Itraconazole^c^		Miconazole^c^	
						Matrix	Sample no.	Formulation	Dose (mg × 2/die)	Time (h)^a^	Volume (mL)	Concentration (μg mL^−1^)	Total amount (mg)	Concentration (μg mL^−1^)	Total amount (mg)
Plasma	1	Capsules	100	4	4400^b^	0.124 (±0.07)	0.546	n.d.	n.d.
	2	Tablets	500	4	4400^b^	n.d.	n.d.	0.508 (±0.05)	2.235
Urine	3	Capsules	100	0	100^d^	n.d.	n.d.	n.d.	n.d.
	4	Capsules	100	3	110^d^	n.d.	n.d.	n.d.	n.d.
	5	Capsules	100	6	100^d^	n.d.	n.d.	n.d.	n.d.
	6	Capsules	100	9	110^d^	n.d.	n.d.	n.d.	n.d.
	7	Capsules	100	12	110^d^	n.d.	n.d.	n.d.	n.d.
	8	Capsules	100	18	110^d^	0.402 (±0.09)	0.044	n.d.	n.d.
	9	Capsules	100	24	110^d^	n.d.	n.d.	n.d.	n.d.
	10	Capsules	100	36	110^d^	n.d.	n.d.	n.d.	n.d.
	11	Tablets	500	0	80^d^	n.d.	n.d.	n.d.	n.d.
	12	Tablets	500	3	380^d^	n.d.	n.d.	n.d.	n.d.
	13	Tablets	500	6	140^d^	n.d.	n.d.	0.136 (±0.08)	0.019
	14	Tablets	500	9	160^d^	n.d.	n.d.	0.229 (±0.04)	0.037
	15	Tablets	500	12	120^d^	n.d.	n.d.	0.102 (±0.07)	0.012
	16	Tablets	500	21	420^d^	n.d.	n.d.	0.098 (±0.06)	0.041
	17	Tablets	500	24	40^d^	n.d.	n.d.	0.048 (±0.03)	0.002
	18	Tablets	500	36	60^d^	n.d.	n.d.	n.d.	n.d.

a Time between the last drug administration and sample collection.

b According to^39^.

c In round brackets are reported standard errors of the mean (SEM) obtained from three independent measures.

d Urine volumes collected from treated patients. n.d.: not detected.

A low concentration of itraconazole was obtained in plasma after 4 h. This value agreed with data previously reported by Hardin et al.^45^, which reported an itraconazole concentration of 0.244 ± 0.090 μg mL^−1^ at 4 h for a single dose of 2 × 100 mg/die per os. The low concentration of itraconazole in the urine suggested that this drug was excreted as non-active metabolites in urine (35%) and faeces (54%)^46^.

The plasma concentration of miconazole partially agreed with previously reported data^46^ for a single dose of 2 × 500 mg/die per os. In fact, a low amount of this analyte (<1%) was excreted unchanged in the urine; while ∼10–20% of this drug was metabolized before excretion^47^. The residual unmodified drug (∼50%) could be eliminated through faeces^48^.

## Conclusions

The MEPS-HPLC-DAD procedure represents a suitable method to analyze simultaneously 12 azole drugs in plasma and urine samples collected from healthy volunteers. The analytical method was optimized using different columns and mobile phase compositions to have a short run time, which can separate several antifungal drugs and benzyl-4-hydroxybenzoate (IS). The best performance of the analytical method was carried out by using a Luna C_18_ column, a binary solvent system made from phosphate buffer (40 mM, pH 2.5) and AcN (58:42, v:v), and a flow rate of 1.0 mL min^−1^. The gradient elution allows separating 12 azole drugs better than iscocratic condition. The performance of MEPS-HPLC-DAD apparatus also depends on pH, ionic strength of the buffer, temperature of the column and solvents. Acid pH (2.5), 40 mM (ionic strength), 25 °C and acetonitrile (solvent elution) provided the best set-up to separate and analyze azole drugs by using a single run of 36 min.

The validation parameters showed that our experimental protocol can be used to detect and quantitate simultaneously bifonazole, butoconazole, clotrimazole, econazole, itraconazole, ketoconazole, miconazole, posaconazole, ravuconazole, terconazole, tioconazole, voriconazole in plasma and urine samples collected from healthy volunteers. Samples from human healthy volunteers after a single oral dose of commercial capsules of itraconazole (100 mg) and tablets of miconazole (500 mg) demonstrated that the selected azole drugs can be analyzed in real biological samples (plasma and urine) and showed analytical parameters similar to standard azole drugs, which are used to develop and validate the method.

No interferences were observed between drugs and biological samples during the validation of method and the analysis of real samples. The MEPS-HPLC-DAD is an easy and quick procedure, which can decrease significantly the variability and the time of the analysis. The MEPS-HPLC-DAD apparatus can provide several advantages for the simultaneous analysis of antifungal drugs in multiple therapy and pharmaceutical science. The MEPS-HPLC-DAD in *off-line* mode can represent an easy and quick analytical tool to separate and analyze several drugs in biological samples without using expensive apparatus and complex methods, which needed skilled operators.

## Supplementary Material

IENZ_1354858_Supplementary_Material.pdf
