# Supplementary material for: Analysis of imidazoles and triazoles in biological samples after MicroExtraction by packed sorbent
Source: J Enzyme Inhib Med Chem. 2017 Aug 4;32(1):1053–63. doi: 10.1080/14756366.2017.1354858 (PMC6010084; doi:10.1080/14756366.2017.1354858)
Supplement: IENZ_1354858_Supplementary_Material.pdf [file IENZ_A_1354858_SM9928.pdf]

Analysis of imidazoles and triazoles in biological samples after MicroExtraction by Packed Sorbent

Cristina Campestre[a], Marcello Locatelli[a,b,*], Paolo Guglielmi[c], Elisa De Luca[a], Giuseppe Bellagamba[a], Sergio Menta[c], Gokhan Zengin[d], Christian Celia[a,e,f], Luisa Di Marzio[a], Simone Carradori[a,*]

[a]University of Chieti – Pescara "G. d'Annunzio"; Department of Pharmacy; via dei Vestini 31; 66100 Chieti; Italy; [b]Interuniversity Consortium of Structural and Systems Biology; Viale Medaglie d'Oro 305; 00136 Roma; Italy. [c]Dipartimento di Chimica e Tecnologie del Farmaco, Sapienza University of Rome, p.le A. Moro 5, 00185 Rome, Italy. [d]Selcuk University; Department of Biology; Konya; Turkey. [e]University of Catanzaro "Magna Græcia"; Inter-Regional Research Center for Food Safety & Health, Viale "S. Venuta"; 88100 Catanzaro; Italy. [f]Houston Methodist Research Institute; Department of Nanomedicine; Houston; Texas 77030; USA.

**Corresponding Authors:*

Marcello Locatelli; Analytical and Bioanalytical Chemistry; University of Chieti – Pescara "G. d'Annunzio"; Department of Pharmacy; Work phone: +39 0871 3554590; Fax: +39 0871 3554911;

E-mail: m.locatelli@unich.it.

Simone Carradori, Medicinal Chemistry, University of Chieti – Pescara "G. d'Annunzio"; Department of Pharmacy; Work phone: +39 0871 3554583; Fax: +39 0871 3554911;

E-mail: simone.carradori@unich.it

Supplementary Material

The Supplementary Material section reports the Ultraviolet/Visible (UV/Vis) spectra and chromatograms, which were collected during the analysis of azole drugs at different maximum wavelengths. The **section S.1** shows the UV/Vis spectra of ketoconazole, terconazole, voriconazole, bifonazole, clotrimazole, tioconazole, econazole, butoconazole, miconazole, posaconazole, ravuconazole, itraconazole and benzyl-4-hydroxybenzoate (IS), respectively. Samples were made in the same mobile phase used for the HPLC analysis. The **section S.2** shows the chromatograms obtained by perfoming the System Suitability Test (SST) analysis of ketoconazole, terconazole, voriconazole, bifonazole, clotrimazole, tioconazole, econazole, butoconazole, miconazole, posaconazole, ravuconazole, itraconazole and benzyl-4-hydroxybenzoate (IS), respectively. The concentration of samples was 4 µg mL^{-1} for different azole drugs and 5 µg mL^{-1} for IS. The mobile phase was used to dissolve azole drugs and IS. The **section S.3** shows the intra-day and inter-day precision (RSD%) and trueness (Bias%) of the analytical method obtained from the analysis of QCs in plasma and urine samples. The **section S.4** shows the physical stability of ketoconazole, terconazole, voriconazole, bifonazole, clotrimazole, tioconazole, econazole, butoconazole, miconazole, posaconazole, ravuconazole and itraconazole, respectively, in plasma and urine samples at different temperatures. The **section S.5** reports the chromatograms of the real samples.

SECTION S.1

The UV/Vis spectra represent the peak of ketoconazole, terconazole, voriconazole, bifonazole, clotrimazole, tioconazole, econazole, butoconazole, miconazole, posaconazole, ravuconazole, itraconazole and benzyl-4-hydroxybenzoate (IS), respectively, at the maximum wavelength, which was used to analyze samples during the HPLC analysis. Azole drugs were dissolved in the same mobile phase used for the HPLC analysis.

KETOCONAZOLE

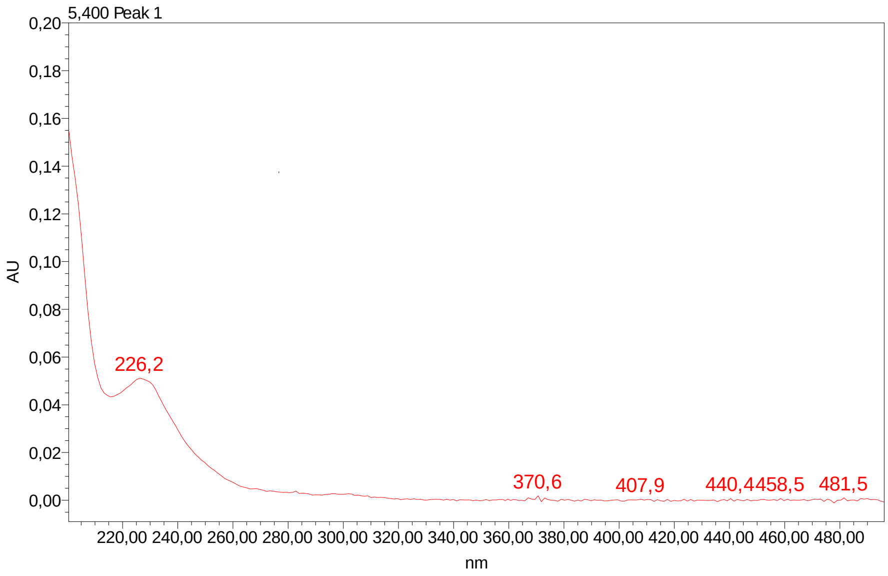

TERCONAZOLE

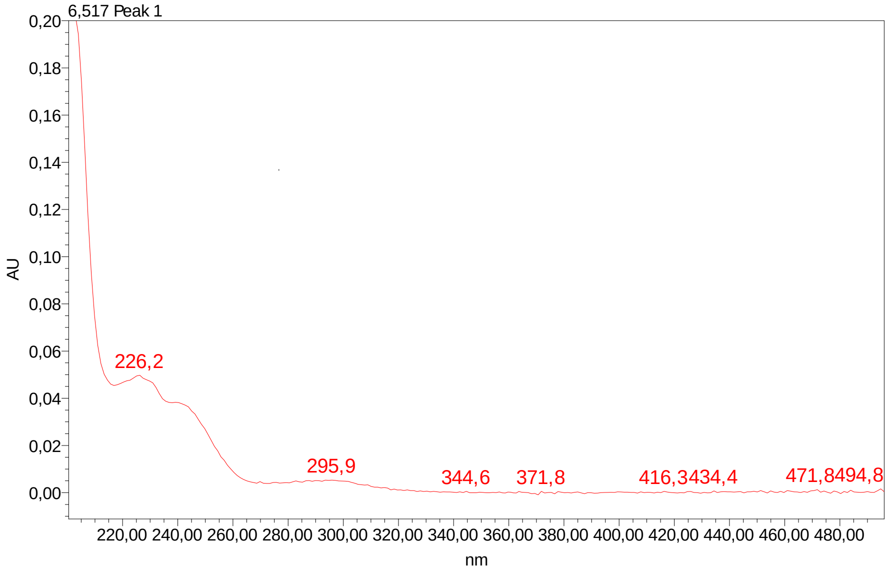

VORICONAZOLE

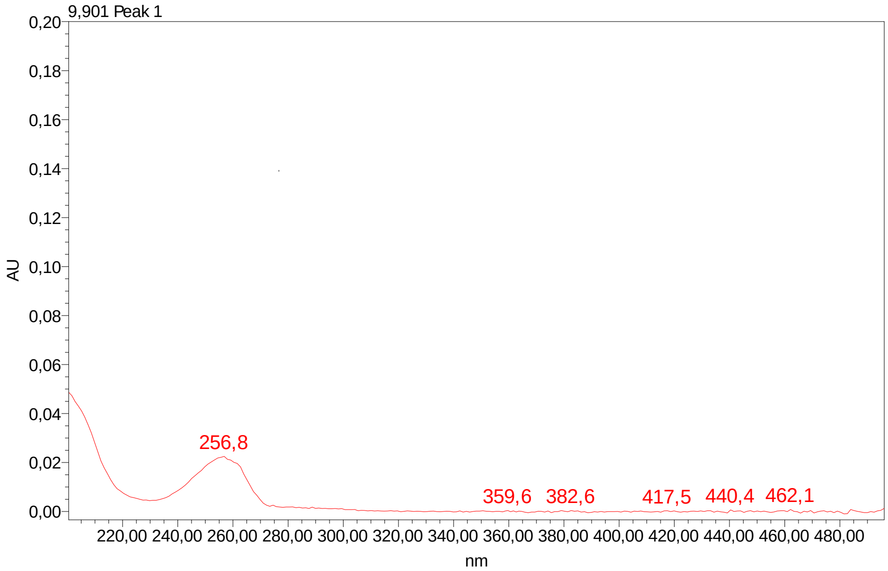

BIFONAZOLE

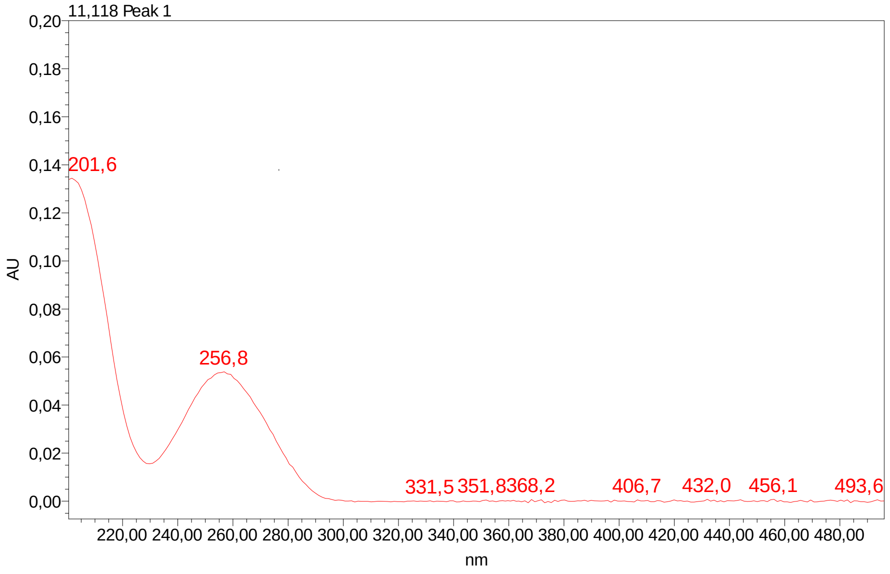

CLOTRIMAZOLE

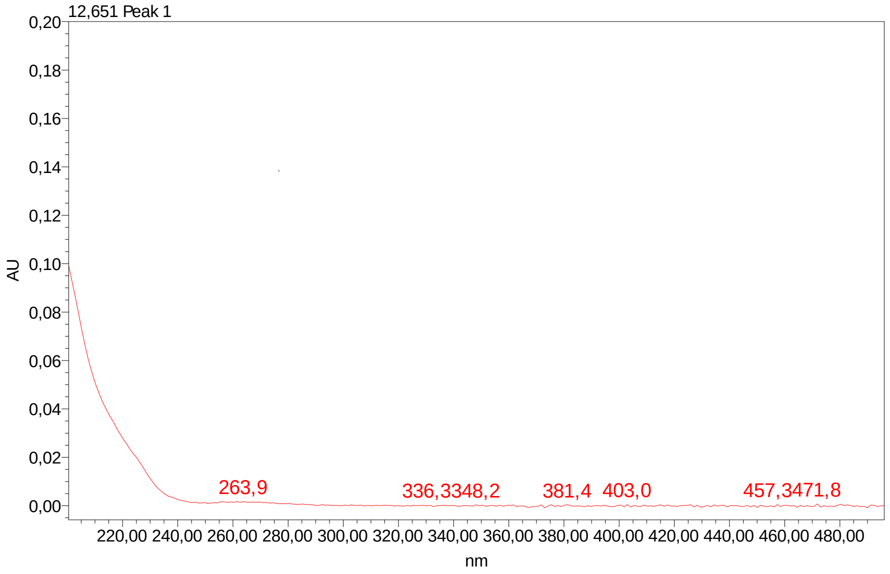

TIOCONAZOLE

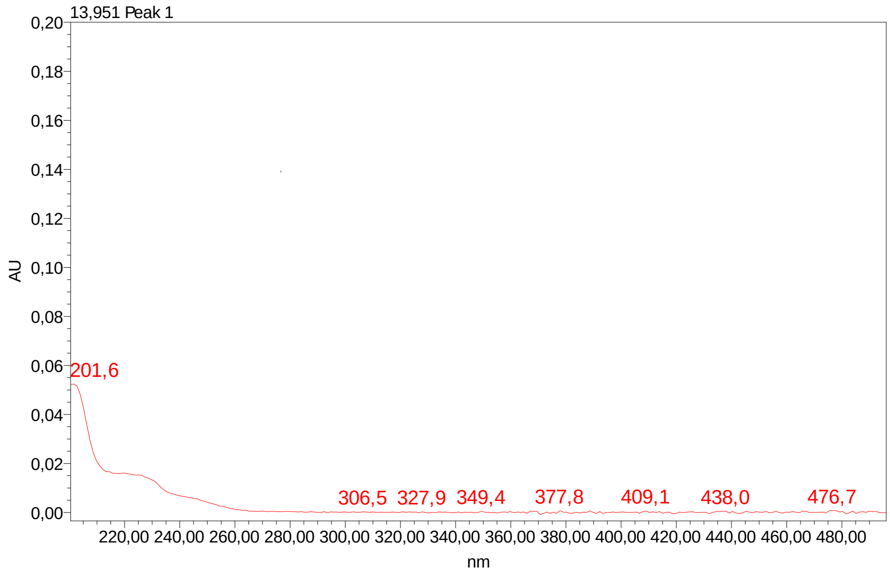

ECONAZOLE

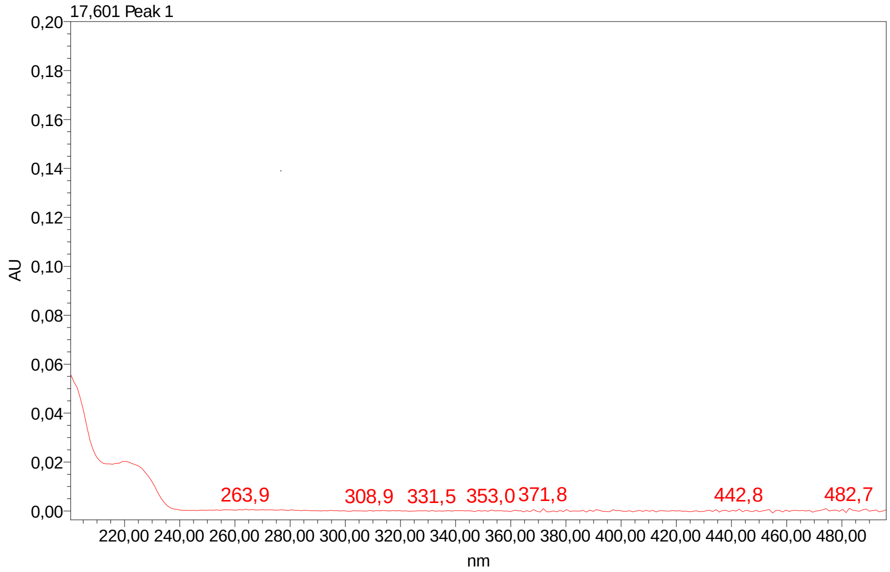

BUTOCONAZOLE

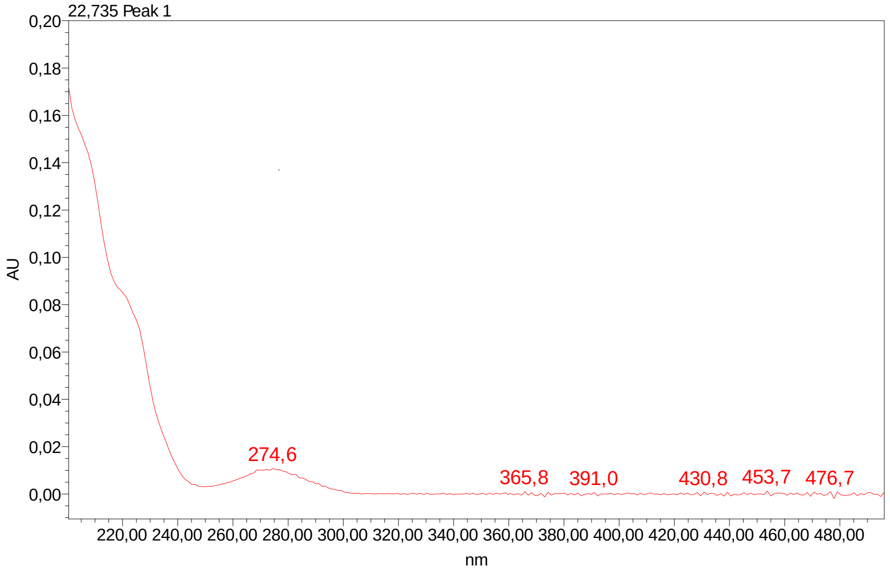

MICONAZOLE

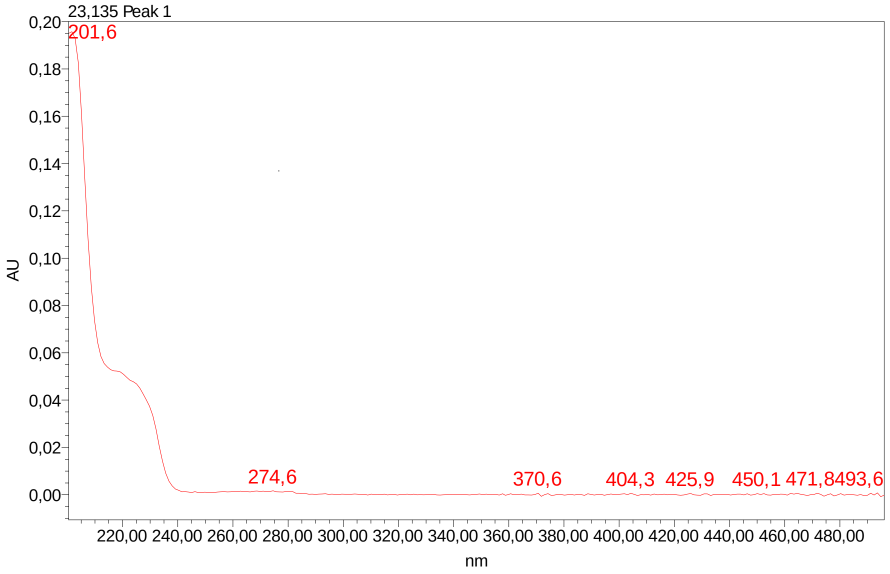

POSACONAZOLE

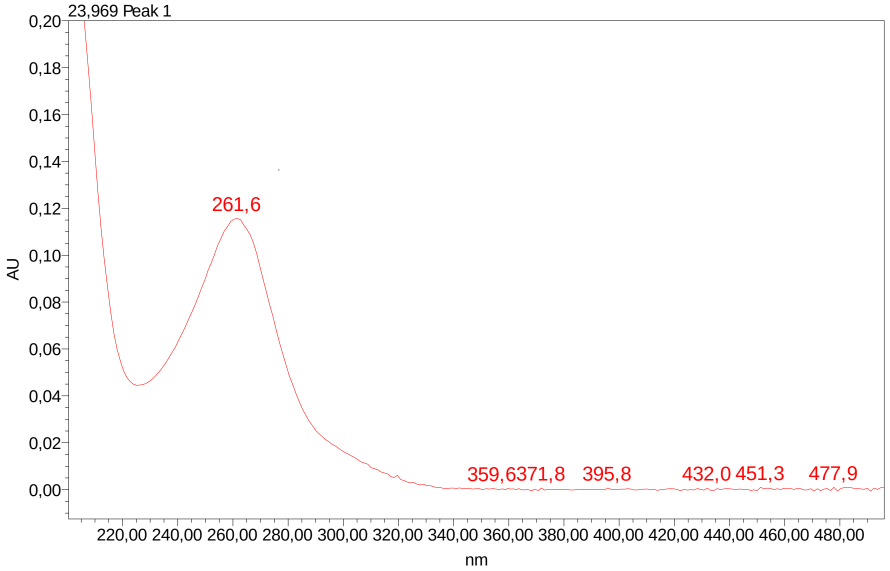

RAVUCONAZOLE

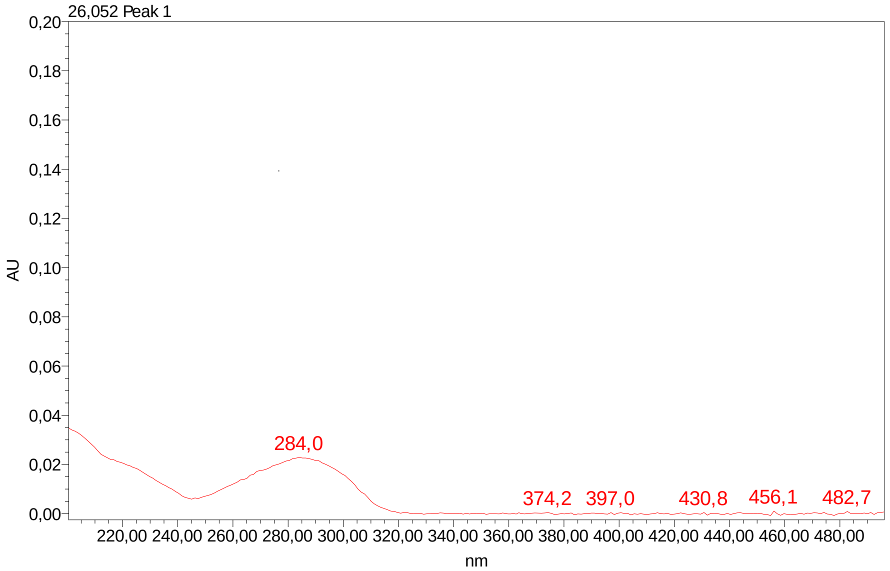

ITRACONAZOLE

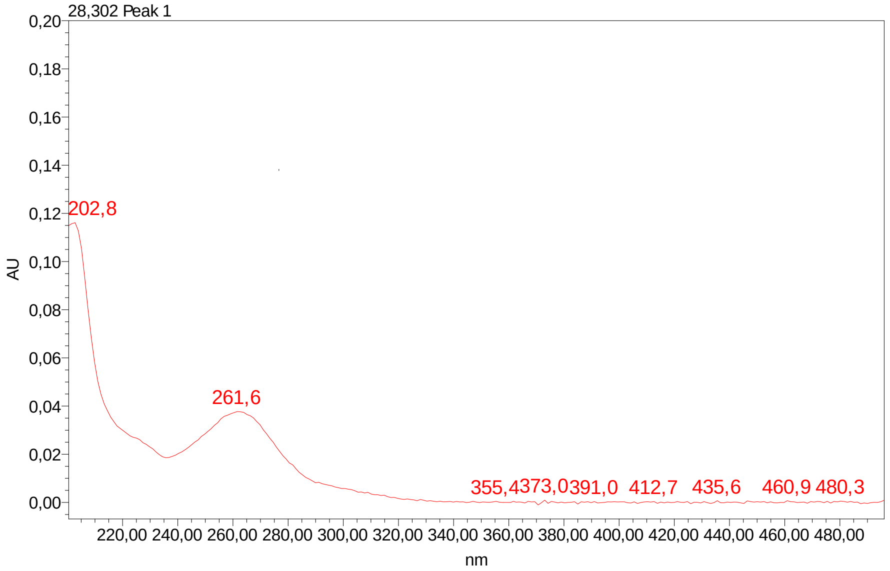

BENZYL-4-HYDROXYBENZOATE (IS)

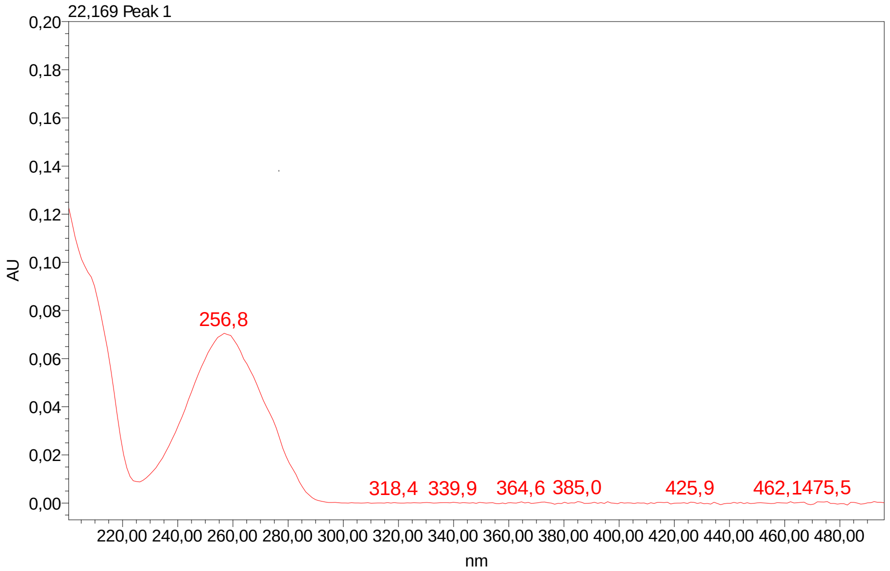

SECTION S.2

The chromatograms were obtained by performing the System Suitability Test (SST) analysis of ketoconazole, terconazole, voriconazole, bifonazole, clotrimazole, tioconazole, econazole, butoconazole, miconazole, posaconazole, ravuconazole, itraconazole and benzyl-4-hydroxybenzoate (IS), respectively. The concentration of samples was 4 μg mL^{-1} for different azole drugs and 5 μg mL^{-1} for IS. The mobile phase was used to dissolve azole drugs and IS. The SST analysis was carried out at 210 nm to separate different azole drugs and benzyl-4-hydroxybenzoate (IS):

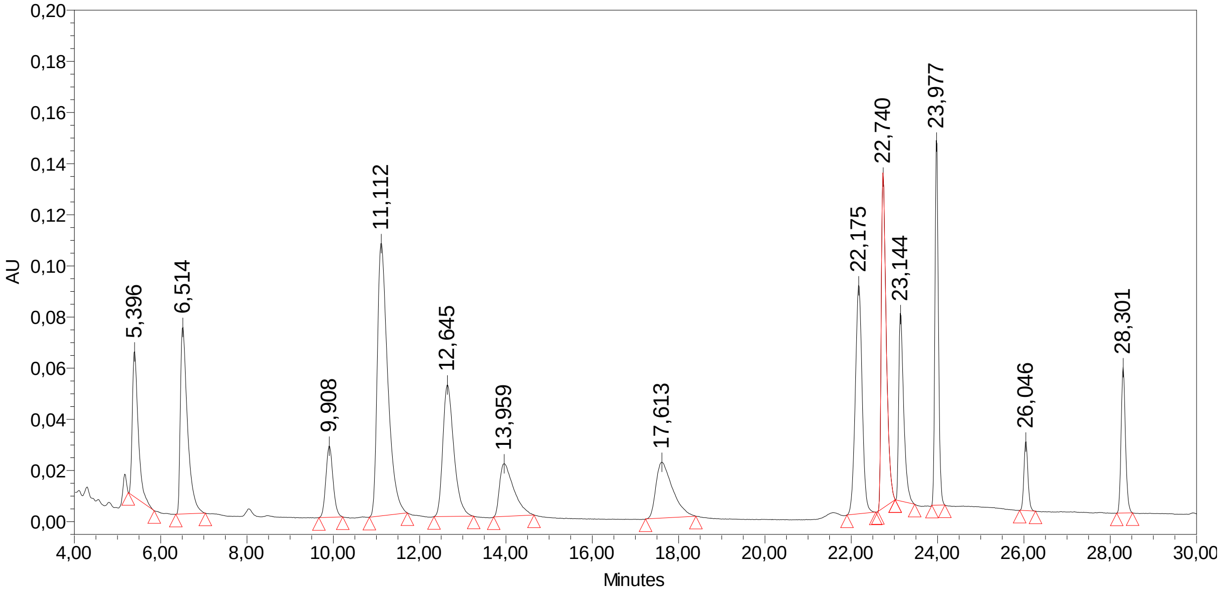

KETOCONAZOLE (retention time of 5.396 min.);

TERCONAZOLE (retention time of 6.514 min.);

VORICONAZOLE (retention time of 9.908 min.);

BIFONAZOLE (retention time of 11.112 min.);

CLOTRIMAZOLE (retention time of 12.645 min.);

TIOCONAZOLE (retention time of 13.959 min.);

ECONAZOLE (retention time of 17.613 min.);

BUTOCONAZOLE (retention time of 22.740 min.);

MICONAZOLE (retention time of 23.144 min.);

POSACONAZOLE (retention time of 23.977 min.);

RAVUCONAZOLE (retention time of 26.046 min.);

ITRACONAZOLE (retention time of 28.301 min.);

BENZYL-4-HYDROXYBENZOATE (IS) (retention time of 22.175 min.).

SECTION S.3

Intra-day and inter-day precision (RSD%) and trueness (Bias%) of the analytical method obtained from the analysis of QCs in plasma and urine samples.

Table S.3.1: Intra-day and inter-day precision (RSD%) and trueness (Bias%) of the analytical method obtained from the analysis of QCs in plasma samples.

		Intra-day			Inter-day		
**Analyte**	**Concentration[a] (µg mL^{-1})**	**Mean back-calculated[a] (µg mL^{-1})**	**BIAS%**	**RSD%**	**Mean back-calculated[a] (µg mL^{-1})**	**BIAS%**	**RSD%**
Ketoconazole	0.4	0.40	0.25	8.93	0.39	-2.26	4.27
Terconazole		0.37	-7.10	6.44	0.41	3.66	9.03
Voriconazole		0.38	-5.38	11.45	0.40	-0.62	9.49
Bifonazole		0.38	-4.04	8.05	0.42	4.48	12.76
Clotrimazole		0.40	1.05	8.91	0.41	3.68	6.29
Tioconazole		0.38	-5.15	12.33	0.41	2.97	5.70
Econazole		0.37	-8.06	3.49	0.41	2.86	13.13
Butoconazole		0.41	1.99	10.67	0.39	-3.27	7.36
Miconazole		0.41	3.15	6.65	0.41	1.34	11.50
Posaconazole		0.38	-5.84	10.03	0.40	0.50	7.38
Ravuconazole		0.42	4.37	8.84	0.43	7.77	6.43
Itraconazole		0.43	8.07	7.81	0.40	-0.96	5.82

Table S.3.1 cont.: Intra-day and inter-day precision (RSD%) and trueness (Bias%) of the analytical method obtained from the analysis of of QCs in plasma samples.

		Intra-day			Inter-day		
**Analyte**	**Concentration[a] (µg mL^{-1})**	**Mean back-calculated[a] (µg mL^{-1})**	**BIAS%**	**RSD%**	**Mean back-calculated[a] (µg mL^{-1})**	**BIAS%**	**RSD%**
Ketoconazole		0.98	-2.20	1.47	0.91	-8.90	8.84
Terconazole		0.99	-1.43	12.04	1.04	4.39	12.11
Voriconazole		0.91	-8.85	6.37	1.02	1.89	4.70
Bifonazole		0.95	-4.85	3.93	0.98	-1.73	6.00
Clotrimazole		0.93	-7.37	6.23	0.97	-2.80	3.73
Tioconazole	1	0.97	-3.15	12.14	0.99	-1.46	11.71
Econazole		0.88	-11.56	2.33	1.01	0.79	11.20
Butoconazole		0.95	-4.57	7.65	0.92	-8.29	4.73
Miconazole		0.90	-10.40	4.70	1.00	0.30	10.16
Posaconazole		0.87	-12.73	1.69	0.98	-1.69	7.93
Ravuconazole		0.94	-5.53	1.57	0.94	-5.64	6.21
Itraconazole		1.07	6.74	4.28	0.95	-4.95	7.87

Table S.3.1 cont.: Intra-day and inter-day precision (RSD%) and trueness (Bias%) of the analytical method obtained from the analysis of of QCs in plasma samples.

		**Intra-day**			**Inter-day**		
**Analyte**	**Concentration[a] (µg mL^{-1})**	**Mean back-calculated[a] (µg mL^{-1})**	**BIAS%**	**RSD%**	**Mean back-calculated[a] (µg mL^{-1})**	**BIAS%**	**RSD%**
Ketoconazole	4	4.26	6.58	3.18	3.99	-0.14	8.19
Terconazole		3.46	-13.44	2.43	3.84	-4.00	4.67
Voriconazole		3.69	-7.68	3.26	4.04	1.09	7.40
Bifonazole		3.81	-4.63	4.02	3.69	-7.75	4.95
Clotrimazole		3.53	-11.66	1.98	3.83	-4.14	5.59
Tioconazole		3.63	-9.31	5.78	4.04	1.11	8.08
Econazole		3.55	-11.20	2.97	3.73	-6.74	7.20
Butoconazole		3.65	-8.80	1.54	3.68	-7.98	3.68
Miconazole		3.78	-5.45	13.00	3.96	-1.11	6.24
Posaconazole		3.89	-2.69	8.72	3.94	-1.62	6.43
Ravuconazole		3.83	-4.32	4.63	3.97	-0.76	7.66
Itraconazole		4.31	7.66	8.00	3.77	-5.83	7.12

The data are the mean values of six experiments (n = 6). [a]Drug concentration is expressed in µg mL^{-1}.

Table S.3.2: Intra-day and inter-day precision (RSD%) and trueness (Bias%) of the analytical method obtained from the analysis of QCs in urine samples.

		Intra-day			Inter-day		
**Analyte**	**Concentration[a] (µg mL^{-1})**	**Mean back-calculated[a] (µg mL^{-1})**	**BIAS%**	**RSD%**	**Mean back-calculated[a] (µg mL^{-1})**	**BIAS%**	**RSD%**
Ketoconazole		0.41	1.97	10.12	0.39	-1.0	9.39
Terconazole		0.36	-9.82	3.46	0.40	0.90	7.34
Voriconazole		0.37	-7.42	3.71	0.42	5.76	3.71
Bifonazole		0.37	-7.12	4.08	0.43	8.03	3.42
Clotrimazole		0.38	-4.68	9.97	0.42	4.90	6.22
Tioconazole	0.4	0.38	-4.70	4.35	0.42	5.43	2.43
Econazole		0.36	-10.15	2.57	0.38	-5.77	6.31
Butoconazole		0.35	-11.54	1.48	0.40	-0.23	10.92
Miconazole		0.35	-11.74	1.76	0.36	-10.23	3.36
Posaconazole		0.35	-12,.	3.67	0.36	-11.06	3.06
Ravuconazole		0.39	-2.81	8.31	0.40	-1.06	4.91
Itraconazole		0.37	-8.15	4.74	0.36	-9.63	3.39

Table S.3.2 cont.: Intra-day and inter-day precision (RSD%) and trueness (Bias%) of the analytical method obtained from the analysis of QCs in urine samples.

		Intra-day			Inter-day		
**Analyte**	**Concentration[a] (µg mL^{-1})**	**Mean back-calculated[a] (µg mL^{-1})**	**BIAS%**	**RSD%**	**Mean back-calculated[a] (µg mL^{-1})**	**BIAS%**	**RSD%**
Ketoconazole	1	1.04	3.95	9.70	1.05	5.38	7.28
Terconazole		1.01	0.65	13.41	1.05	4.70	4.43
Voriconazole		1.06	6.23	9.19	1.12	12.40	1.57
Bifonazole		1.07	6.82	4.79	1.01	1.19	6.45
Clotrimazole		1.03	3.00	3.53	1.02	2.41	3.35
Tioconazole		1.00	0.07	5.30	1.11	11.04	4.03
Econazole		0.99	-0.99	7.57	1.00	-0.11	4.29
Butoconazole		1.06	6.08	6.27	1.07	6.83	4.31
Miconazole		1.02	2.00	6.38	1.04	3.80	3.32
Posaconazole		1.10	9.60	1.22	1.06	5.97	5.12
Ravuconazole		1.05	5.05	9.70	0.92	-8.02	6.29
Itraconazole		1.09	8.75	5.51	1.06	5.82	6.63

Table S.3.2 cont.: Intra-day and inter-day precision (RSD%) and trueness (Bias%) of the analytical method obtained from the analysis of QCs in urine samples.

		Intra-day			Inter-day		
**Analyte**	**Concentration[a] (µg mL^{-1})**	**Mean back-calculated[a] (µg mL^{-1})**	**BIAS%**	**RSD%**	**Mean back-calculated[a] (µg mL^{-1})**	**BIAS%**	**RSD%**
Ketoconazole	4	4.34	8.58	5.01	3.76	-5.90	8.22
Terconazole		4.46	11.59	1.82	3.65	-8.83	5.59
Voriconazole		4.39	9.86	4.89	3.64	-9.10	4.59
Bifonazole		4.31	7.82	3.13	3.99	-0.33	7.41
Clotrimazole		4.32	7.91	3.03	3.73	-6.83	5.57
Tioconazole		4.36	8.90	3.88	3.63	-9.19	7.11
Econazole		4.28	6.92	3.22	3.79	-5.20	5.47
Butoconazole		4.35	8.81	2.76	3.78	-5.62	7.15
Miconazole		3.91	-2.29	2.36	3.84	-4.05	4.55
Posaconazole		3.75	-6.27	4.30	3.68	-8.02	5.56
Ravuconazole		4.34	8.54	4.08	4.21	5.20	7.68
Itraconazole		4.32	7.90	3.01	3.80	-5.10	2.35

The data are the mean values of six experiments (n = 6). [a]Drug concentration is expressed in µg mL^{-1}.

SECTION S.4

The physical stability of twelve azole drugs and IS in plasma and urine samples was carried out at different storage conditions. Samples were stable for long time when the mean concentration of three stored QCs did not show a variation of ±15% for its nominal concentration under the storage condition. The QCs of stored samples at room temperature and + 4 °C were obtained using a new calibration curve and compared to the nominal initial concentrations of azole drugs. Results of injected samples were integrated using the new curve without showing any significant difference for the integrated peak of samples. The stability of stock and working solutions in mobile phase was accepted for a mean response of the test solution, which did not show a variation below 5.0% compared to the mean response of the control solution.

Table S.4.1: Physical stability of ketoconazole, terconazole, voriconazole, bifonazole, clotrimazole, tioconazole, econazole, butoconazole, miconazole, posaconazole, ravuconazole, and itraconazole, respectively, in plasma at different temperatures and storage conditions.

	Ketoconazole	Terconazole	Voriconazole	Bifonazole	Clotrimazole	Tioconazole	Condition
QC low			**0.4**				**SHORT TERM STABILITY** **Room temperature (25 °C ± 1 °C)**
Time = 24 hours	95.2	91.6	98.3	92.7	107.2	99.0	
QC medium			**1.0**				
Time = 24 hours	99.0	107.0	106.8	100.2	101.7	95.9	
QC high			**4.0**				
Time = 24 hours	94.6	107.5	103.8	103.5	102.7	98.2	
QC low			**0.4**				**LONG TERM STABILITY** **(4 °C ± 1 °C)**
Time = 30 days	107.9	105.0	101.6	97.7	97.8	86.4	
QC medium			**1.0**				
Time = 30 days	100.8	103.5	97.3	102.0	102.2	94.2	
QC high			**4.0**				
Time = 30 days	93.6	102.0	93.0	106.2	106.5	102.1	
QC low			**0.4**				**LONG TERM STABILITY** **(-20 °C ± 1 °C)**
Time = 30 days	98.8	96.3	94.8	109.8	92.5	108.9	
QC medium			**1.0**				
Time = 30 days	105.4	96.1	94.5	91.3	95.3	103.0	
QC high			**4.0**				
Time = 30 days	94.1	107.7	95.9	106.4	106.5	97.4	
QC low			**0.4**				**3 FREEZE/THAW CYCLES**
Time = 30 days	100.7	100.8	105.0	110.4	101.8	107.5	
QC medium			**1.0**				
Time = 30 days	96.9	103.8	94.8	90.3	90.0	93.0	
QC high			**4.0**				
Time = 30 days	99.4	106.8	96.3	108.3	106.6	101.1	

Data are expressed as the percentage of different values with respect to the starting drug concentration at the time t_0.

Table S.4.1 cont.: Physical stability of ketoconazole, terconazole, voriconazole, bifonazole, clotrimazole, tioconazole, econazole, butoconazole, miconazole, posaconazole, ravuconazole, and itraconazole, respectively, in plasma at different temperatures and storage conditions.

	Econazole	Butoconazole	Miconazole	Posaconazole	Ravuconazole	Itraconazole	Condition	Stability
QC low				**0.4**			Room temperature (25 °C ± 1 °C)	SHORT TERM STABILITY
Time = 24 hours	99.7	98.3	100.5	93.9	99.6	104.6		
QC medium				**1.0**				
Time = 24 hours	102.3	100.5	101.8	100.0	93.5	99.1		
QC high				**4.0**				
Time = 24 hours	101.6	101.9	95.0	80.5	107.7	102.5		
QC low				**0.4**			(4 °C ± 1 °C)	LONG TERM STABILITY
Time = 30 days	99.4	109.0	105.2	104.7	95.1	109.4		
QC medium				**1.0**				
Time = 30 days	99.7	105.6	104.1	99.1	99.3	101.2		
QC high				**4.0**				
Time = 30 days	100.0	102.3	102.9	93.5	103.4	93.0		
QC low				**0.4**			(-20 °C ± 1 °C)	
Time = 30 days	103.0	108.3	98.3	943	106.5	107.6		
QC medium				**1.0**				
Time = 30 days	90.4	90.6	93.5	96.3	91.0	89.2		
QC high				**4.0**				
Time = 30 days	111.5	107.3	100.4	95.7	86.0	92.6		
QC low				**0.4**				3 FREEZE/THAW CYCLES
Time = 30 days	103.8	105.9	99.8	94.3	107.9	96.9		
QC medium				**1.0**				
Time = 30 days	92.0	92.8	95.5	90.1	99.3	102.5		
QC high				**4.0**				
Time = 30 days	102.9	106.1	104.4	94.6	95.3	102.7		

Data are expressed as the percentage of different values with respect to the starting drug concentration at the time t_0.

Table S.4.2: Physical stability of ketoconazole, terconazole, voriconazole, bifonazole, clotrimazole, tioconazole, econazole, butoconazole, miconazole, posaconazole, ravuconazole, and itraconazole, respectively, in urine at different temperatures and storage conditions.

	Ketoconazole	Terconazole	Voriconazole	Bifonazole	Clotrimazole	Tioconazole		
QC low			**0.4**				**Room temperature (25 °C ± 1 °C)**	**SHORT TERM STABILITY**
Time = 24 hours	86.7	102.0	105.5	99.4	81.1	107.1		
QC medium			**1.0**					
Time = 24 hours	84.8	110.6	116.0	92.2	109.5	85.0		
QC high			**4.0**					
Time = 24 hours	110.2	97.8	111.4	101.5	110.2	98.8		
QC low			**0.4**				**(4 °C ± 1 °C)**	**LONG TERM STABILITY**
Time = 30 days	83.4	98.2	97.0	100.3	87.1	85.1		
QC medium			**1.0**					
Time = 30 days	85.5	113.0	110.1	105.5	111.8	84.7		
QC high			**4.0**					
Time = 30 days	84.9	94.7	89.0	98.6	97.2	89.6		
QC low			**0.4**				**(-20 °C ± 1 °C)**	
Time = 30 days	105.7	97.8	103.7	99.8	82.8	86.9		
QC medium			**1.0**					
Time = 30 days	92.2	107.4	111.7	107.7	97.2	82.3		
QC high			**4.0**					
Time = 30 days	80.1	89.5	86.3	92.3	87.4	90.7		
QC low			**0.4**					**3 FREEZE/THAW CYCLES**
Time = 30 days	79.9	77.8	102.4	97.8	91.5	77.8		
QC medium			**1.0**					
Time = 30 days	91.4	89.5	102.1	89.1	98.2	75.4		
QC high			**4.0**					
Time = 30 days	84.7	92.3	88.8	98.4	95.1	87.9		

Data are expressed as the percentage of different values with respect to the starting drug concentration at the time t_0.

Table S.4.2 cont.: Physical stability of ketoconazole, terconazole, voriconazole, bifonazole, clotrimazole, tioconazole, econazole, butoconazole, miconazole, posaconazole, ravuconazole, and itraconazole, respectively, in urine at different temperatures and storage conditions.

	Econazole	Butoconazole	Miconazole	Posaconazole	Ravuconazole	Itraconazole		
QC low				**0.4**			**Room temperature (25 °C ± 1 °C)**	**SHORT TERM STABILITY**
Time = 24 hours	91.2	88.2	105.9	94.0	99.8	107.5		
QC medium				**1.0**				
Time = 24 hours	96.7	99.8	91.9	100.3	104.7	89.0		
QC high				**4.0**				
Time = 24 hours	102,8	99.6	88,7	95.2	105.1	87.5		
QC low				**0.4**			**(4 °C ± 1 °C)**	**LONG TERM STABILITY**
Time = 30 days	96.2	88.5	103.7	81.8	103.6	90.3		
QC medium				**1.0**				
Time = 30 days	100.6	90.7	79.1	95.8	97.2	85.8		
QC high				**4.0**				
Time = 30 days	86.7	82.2	89.6	78.0	102.9	100.2		
QC low				**0.4**			**(-20 °C ± 1 °C)**	
Time = 30 days	101.6	95.2	100.8	84.3	93.4	107.9		
QC medium				**1.0**				
Time = 30 days	96.7	82.6	88.4	96.6	108.2	88.1		
QC high				**4.0**				
Time = 30 days	76.2	92.5	101.4	85.8	101.7	95.4		
QC low				**0.4**				**3 FREEZE/THAW CYCLES**
Time = 30 days	100.4	97.7	85.9	94.3	104.1	73.5		
QC medium				**1.0**				
Time = 30 days	87.4	78.3	91.7	82.9	90.2	83.0		
QC high				**4.0**				
Time = 30 days	90.9	96.2	84.8	86.3	100.8	96.3		

Data are expressed as the percentage of different values with respect to the starting drug concentration at the time t_0.

SECTION S.5

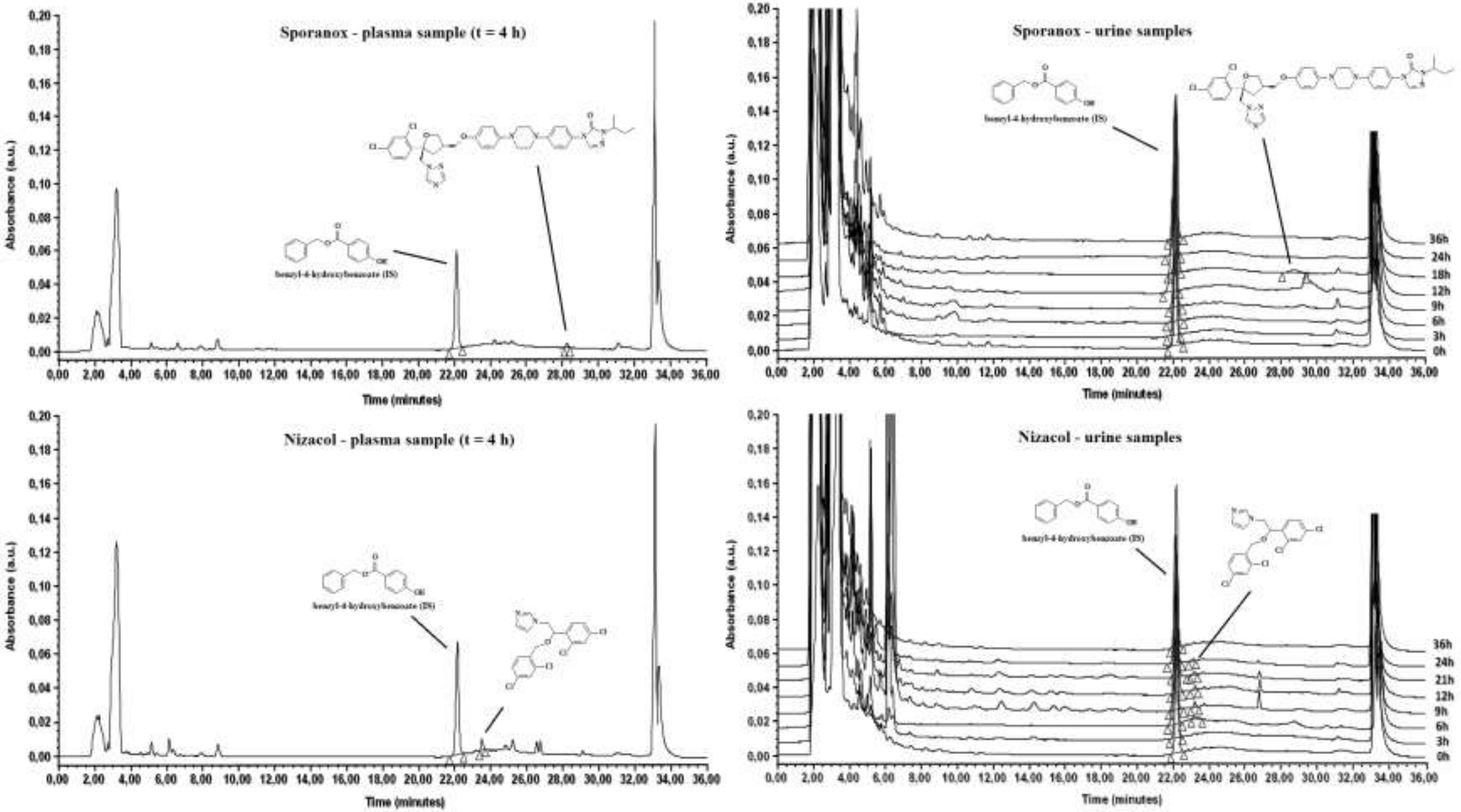

Figure S.5.1. Chromatograms of itraconazole and miconazole extracted from real samples. Sporanox® (100 mg × 2/die per os) was detected at the wavelength of 210 nm (up); Nizacol® (500 mg × 2/die per os) was detected at the wavelength of 210 nm (bottom). Left: plasma samples collected from healthy human volunteers after 4 h, right: urine samples collected from human volunteers at different times as reported in Table 4 (main text).

177
